# The Cost of Provisions in Institutions.—III

**Published:** 1904-04-23

**Authors:** 


### HOSPITAL ADMINISTRATION.

### CONSTRUCTION AND ECONOMICS.

### THE COST OF PROVISIONS IN INSTITUTIONS.—III.

By the Author of "Hospital Expenditure—The Commissariat."

(Continued from page 50.)

THE ROYAL FREE HOSPITAL (165 Available Beds).

The cost of provisions per bed at the above hospital during the last five years is as under;—

| | £ | Average of Occupied Beds. |
|---|---|---|
| 1902 ... ... ... | 28 | 144 |
| 1901 ... ... ... | $26\frac{3}{4}$ | 131 |
| 1900 ... ... ... | 27 | 120 |
| 1899 ... ... ... | $28\frac{1}{4}$ | 127 |
| 1898 ... ... ... | $24\frac{3}{4}$ | 143 |

Together with the board of patients the figures represent the cost of boarding the medical staff, nursing staff, and all other members of the household.

The following table shows the average cost of provisions for the hospital during the last five years, when the board of the patients, estimated at 6s. 7d. a week, or £17 a year, has been deducted. The cost of the patients is based on the average of occupied beds; that of the household on the number of available beds also, viz., 170:—

| Year. | No. of occupied beds out of 165 available. | Total cost of provisions. | Cost of patients at £17 a year. | Average cost of provisions exclusive of patients | |
|---|---|---|---|---|---|
| | | | | Per available bed. | Per occupied bed. |
| | | £ | £ | £ | £ |
| 1902 ... | 144 | 4,090 | 2,448 | 10 | $11\frac{1}{4}$ |
| 1901 ... | 131 | 3,565 | 2,227 | 8 | 10 |
| 1900 ... | 120 | 3,282 | 2,040 | $7\frac{1}{2}$ | $10\frac{1}{4}$ |
| 1899 ... | 127 | 3,598 | 2,159 | $8\frac{3}{4}$ | $11\frac{1}{4}$ |

It will be noticed that the fluctuations in cost when the variable element of the patient is deducted are much the same as when the cost of provisions is reckoned in the usual way on the basis of the number of occupied beds. It is not a wide fluctuation, being limited to a range in both cases of from £3 to £$3\frac{1}{2}$. The highest and lowest averages per occupied bed (£$28\frac{1}{4}$ and £$24\frac{3}{4}$) occur in years when the number of occupied beds was 144 and 127 respectively. The reason of this uniformity of expenditure may be found in the fact that the proportionate expense of the patients food in this hospital works out at a high rate as compared with that of the rest of the household; or, to put it in another way, the cost of boarding inmates other than patients does not overbalance it, as is sometimes the case.

| — | Number | Average number of available beds to each | Average of occupied beds to each |
|---|---|---|---|
| Medical and other officers | $5\frac{1}{2}$ | 31 | 26 |
| Miscellaneous (about 23 daily) ... ... | $11\frac{1}{2}$ | — | — |
| Nursing staff ... ... | 49 | 3·4 | 2·9 |
| Male servants ... ... | 4 | 41 | 36 |
| Female servants including two laundry-maids ... ... | 11 | 15 | 13 |
| Total (a rough estimate) | 81 | 2 | 1·8 |

It will be interesting to note the number for 1902. Partial board reckons as half. Luncheon only, one-third.

It has not been easy to arrive at a very accurate computation of those boarded in the hospital, as the number of persons for whom lunch is provided varies from day to day. It is believed, however, that the above figures are substantially correct, those boarded in the hospital other than patients being 35 per cent. of the total number of inmates.

We proceed to examine the cost of each item of provision as enumerated in the published accounts for 1902.

Daily average of patients, 144
Daily average of non-patients, 81
Daily average boarded in hospital, 225.

| — | Total cost | Cost per occupied bed | | Cost per head for all inmates | |
|---|---|---|---|---|---|
| | £ | £ | s. | £ | s. |
| Meat ... ... | 1,058 | 7 | 7 | 4 | 14 |
| Fish and poultry ... | 663 | 4 | 12 | 3 | 0 |
| Butter, cheese, etc. | 563 | 3 | 18 | 2 | 15 |
| Eggs ... ... | 213 | 1 | 9 | 0 | 19 |
| Milk ... ... | 707 | 4 | 18 | 3 | 3 |
| Bread, flour, etc. ... | 230 | 1 | 12 | 1 | 0 |
| Groceries ... ... | 409 | 2 | 16 | 1 | 16 |
| Vegetables ... ... | 209 | 1 | 9 | 0 | 19 |
| Malt liquors ... | 34 | 0 | 4 | 0 | 3 |
| Ice and mineral waters ... ... | 73 | 0 | 10 | — | |
| Wine and spirits ... | 68 | 0 | 9 | — | |

The exact cost of boarding each of the four divisions of the household—officers, nurses, household, patients—is accurately separated in the accounts, together with the amounts required under each heading week by week. Every week a report is presented to the board, showing the weekly cost of victualling the hospital under these headings. And in this way any want of control, such as may be shown, for instance, by a new housekeeper, is at once apparent. Of the three strands which, as we have said before, go to make up perfect economy, viz., prices, numbers, distribution, it is the third which is the hardest to control It is this third strand, whether it be called distribution, management, or care, which is obtained by this continual observation of the weekly average. Hence it has been ascertained that a change of housekeeper will suffice to make a difference of about 1s. a head in the weekly expenditure without any change being perceptible in the quality of the meals. Now the difference of 1s. a week will amount in the course of a year, for 100 persons, to as much as £260. Does it not seem worth while to employ a clerk one day a week in making the rather fastidious calculations necessary to this system, in order to check from the outset any such genial relaxation from the laws of rigid economy?

In a given week the patients required 460 lb. of meat, including gravy beef. This gives an average of a little over 3 lb. a head. If the patients on exclusively milk diet be omitted, and on an average there are found to be about 11 receiving neither meat nor beef-tea, the average consumption per patient of uncooked meat, as received from the butcher, is exactly $3\frac{1}{2}$ lb. a week.

The average consumption of meat by the nursing staff was exactly 5 lb. a week, not, of course, including bacon, fish, etc.

As regards milk, the average quantity consumed by the patients amounted in a given week to 8·7 quarts, or $2\frac{1}{2}$ pints a day.

The nursing staff, among whom milk is much used as a beverage, used at the rate of four quarts a week each, or rather over a pint a day.

The diet table for patients is as follows:—

| *Ordinary diet.* | *Light diet.* |
|---|---|
| Meat: Men, 4 ozs. | Fish, 10 ozs., or |
| Women, 3 ozs. | Minced meat, 4 ozs., or |
| Children, 2 ozs. | Beef tea, 1 pint. |
| Bread: Adults, 10 ozs. | Bread, 10 ozs. |
| Children, 8 ozs. | Potatoes, 6 ozs. |
| Potatoes, 6 ozs. | Pudding. |
| Green vegetables twice a week. | Milk, 2 pints. |
| Pudding. | |
| Milk, 1 pint. | |
| Soup, $\frac{1}{2}$ pint. | |

Tea, sugar and butter are provided for all patients. Fluid diet consists of 4 pints of milk, or 3 pints of milk with 1 of beef tea. The usual "extras" can be ordered on special order forms which must be signed by the senior resident medical officer.

The average weekly cost of the patient in 1902 was 6s. 7d. as against 6s. 9d. in 1903. The average weekly cost of the nurses in 1902 was 7s. 4d., in 1903 7s. 7d. There is one table for the nursing staff, matron, sisters and nurses dining together and practically otherwise living alike.

The cost of the medical officers varied in like manner between 19s. 4d. and £1 0s. 3d. a week.

We hope later on to present our readers with a synopsis of the system of accounts by which this careful and valuable estimate of expenditure is obtained.

